# Diagnostic Model Development for IC/BPS and Its Subtypes Using Clinical Indicators, Urinary Biomarkers, and Single‐Cell Transcriptomic Analysis

**DOI:** 10.1096/fj.202602743R

**Published:** 2026-08-04

**Authors:** Cheng Luo, Yonghui Guan, Yifala Dilimulati, Xinping Zhang, Cancan Wang, Mulati Rexiati

**Affiliations:** ^1^ Department of Urology Surgery The First Affiliated Hospital of Xinjiang Medical University Urumqi Xinjiang China

**Keywords:** diagnostic model, inflammatory response, interstitial cystitis/bladder pain syndrome, machine learning, single‐cell RNA sequencing, tumor necrosis factor‐α

## Abstract

Interstitial cystitis/bladder pain syndrome (IC/BPS) is a chronic, heterogeneous, and often debilitating condition. This condition is often misdiagnosed, as it overlaps significantly with other urinary infections, necessitating the development of new markers and targets to improve diagnosis and therapy. Tumor necrosis factor‐α (TNF‐α) participates in IC/BPS inflammation, but its role remains understudied. This study aimed to develop a diagnostic model for IC/BPS and its subtypes to uncover underlying mechanisms, new biomarkers, and TNF‐α‐targeted treatments. Patients with non‐Hunner IC (NHIC, *n* = 196), Hunner IC (HIC, *n* = 56), and healthy controls (HC, *n* = 230) were enrolled in the study. A diagnostic model was developed based on key features identified by SHapley Additive exPlanations (SHAP) analysis. Molecular analysis using Gene Expression Omnibus (GEO) data and single‐cell RNA sequencing (scRNA‐seq) showed cellular heterogeneity and inflammatory networks. In silico TNF‐α knockout was performed using scTenifoldKnk to investigate the function of TNF‐α. Both NHIC and HIC subtypes present with severe clinical symptoms and dysregulated urinary biomarker profiles, with the HIC subtype showing significantly more severe symptoms and markedly increased levels of specific inflammatory mediators. A total of 127 machine learning (ML) models were constructed, of which most reached an AUC of 1.000 in training and external validation cohorts. Lasso+plsRglm was identified as the optimal diagnostic model. Core diagnostic features included nocturia frequency (NUF), visual analogue scale (VAS), functional bladder wall thickness (BWT), and TNF‐α. Transcriptomics identified inflammation‐related patterns and pathways between IC/BPS subtypes. scRNA‐seq identified 12 cell types and an elevated inflammatory state. We discovered a TNF‐α‐driven inflammatory network influencing intergroup heterogeneity of IC/BPS, identified candidate diagnostic biomarkers to differentiate IC/BPS from HC, and highlighted possible therapeutic targets, thereby laying the groundwork for future clinical translation research.

## Graphical abstract

Subjects were divided into HC, NHIC, and HIC groups. Clinical Characteristics, urinary ultrasound parameters, and urinary biomarkers were collected for ML screening of predictive signatures. DEGs are identified from GEO transcriptomic data; scRNA‐seq delineates cell clusters, pseudotime trajectories and intercellular communication. Virtual gene knockout validated the function of TNF‐α.

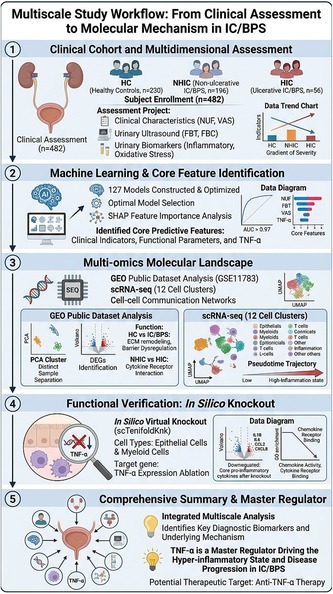

## 1 Introduction

Interstitial cystitis/bladder pain syndrome (IC/BPS) is a chronic, inflammatory pelvic condition that causes pelvic pain, urine frequency, urgency, and nocturia, with symptoms continuing for more than 6 weeks [1, 2]. The incidence rate of this condition has been on the rise globally, with the female population constituting over 95% of cases [3]. While the pathophysiology of IC/BPS is complex and not well studied, it is currently known to be closely linked to several pathological mechanisms that include immune dysregulation, impaired bladder epithelial barrier function, abnormal inflammatory responses, disrupted oxidative stress balance, and progressive fibrosis [4]. The effective management of IC/BPS in clinical practice is particularly limited by a lack of specific diagnostic biomarkers, which leads to a diagnosis dependent on symptom evaluation, imaging examinations, and exclusionary diagnostic strategies. As a result, IC/BPS is often misdiagnosed as bladder stones, urinary tract infections, and other conditions, which results in consistently high rates of misdiagnosis and missed diagnosis [5]. Thus, the development of new treatment targets, the identification of specific diagnostic biomarkers, and the exploration of the fundamental pathogenic mechanisms of IC/BPS are key factors for improving the management of this condition.

It is important to note that significant pathophysiological heterogeneity is observed in IC/BPS. Its categories [non‐Hunner type‐IC (NHIC) and Hunner‐type IC (HIC)] are based on cystoscopic findings. These subtypes clinically manifest differently and respond differently to treatment [6]. Previous studies report that numerous inflammatory mediators are abnormally overexpressed in the urine and bladder tissue of IC/BPS patients. Among these, the canonical pro‐inflammatory cytokine, tumor necrosis factor‐α (TNF‐α), initiates a cascade of inflammatory molecules, such as interleukin‐6 (IL‐6) and interleukin‐8 (IL‐8), by activating the downstream NF‐κB signaling pathway. TNF‐α increases the production of enzymes linked to oxidative stress, which then aggravates tissue fibrosis and damages the bladder epithelial barrier, creating a vicious cycle of inflammation, injury, and fibrosis [7]. Additionally, a significantly high infiltration of immune cells occurs in the bladder tissue of IC/BPS patients, including neutrophils, myeloid cells, and activated immune cells. These cells facilitate the advancement of the disease by secreting inflammatory cytokines and modulating immunological regulatory mechanisms [8]. Nevertheless, the inability of conventional bulk sequencing and conventional molecular biological techniques to accurately dissect cellular heterogeneity and intercellular communication networks within the bladder microenvironment in IC/BPS has made it challenging to identify key regulatory targets underlying disease pathogenesis.

The widespread implementation of precision medicine and the rapid progress achieved by artificial intelligence have enabled the development of novel research strategies. This has facilitated the advancement of powerful clinical data‐driven models that can precisely diagnose diseases and conduct mechanistic investigations. Machine learning (ML) models developed by combining multidimensional data such as clinical symptoms, imaging features, and circulating biomarkers can lead to early prediction of diseases and stratification of their subtypes. Furthermore, essential pathological characteristics can also be identified [9]. The SHapley Additive exPlanations (SHAP) algorithm, based on game theory, quantifies the contribution of individual features to model predictions, allowing the translational application of clinical diagnostic models [10]. In addition, the development of single‐cell sequencing technologies has transformed the analysis of cellular heterogeneity in disease by enabling accurate single‐cell characterization of target‐expressing cell populations, interrogation of cell–cell communication networks, and the exploration of gene regulatory circuits [11]. The combined use of these technologies consequently offers great opportunities to systematically characterize the pathophysiology of IC/BPS and develop a robust, precise framework for accurate clinical diagnosis.

Our study aims to address the current research gaps in IC/BPS clinical diagnosis, management, and mechanistic research by building on recent technology advancements. We determined key diagnostic biomarkers and investigated the molecular mechanism driving IC/BPS using a comprehensive, multi‐stage study design. Eligible study subjects with NHIC and HIC, as well as healthy controls (HC), were enrolled. Their clinical characteristics, urinary ultrasound parameters, and urinary biomarkers were systematically measured. A multi‐dimensional analytical framework was developed using these clinical datasets, and 127 ML models were constructed and optimized to screen key predictive variables to diagnose IC/BPS and accurately categorize its subtypes, resulting in the development of a successful diagnostic model. Furthermore, public transcriptomic data from the Gene Expression Omnibus (GEO) database were examined to identify differentially expressed genes (DEGs) and functional pathways, and single‐cell RNA sequencing (scRNA‐seq) was used to define cellular heterogeneity, developmental trajectories, and cell–cell communication networks in IC/BPS. An analysis of virtual gene knockout was also conducted to verify the regulatory role of the key target gene TNF‐α, thereby uncovering the pathophysiology of IC/BPS and possible treatment targets. This research approach combines clinical diagrams, mechanistic analysis, and drug target identification to offer robust theoretical and experimental support for effective clinical translation of IC/BPS research. Our work may offer great potential for scientific significance and clinical impact.

## 2 Materials and Methods

### 2.1 Study Subjects

Patients diagnosed with IC/BPS who received treatment at the Department of Urology in our hospital from January 2021 to December 2025 were enrolled as the case group. Healthy volunteers without urinary system diseases who underwent physical examination during the same period were recruited as the HC group. All participants voluntarily participated in the study and cooperated with data collection and relevant examinations. This study was approved by the Medical Ethics Committee of Xinjiang Medical University (Approval No.: K202511‐18). Written informed consent was obtained from each participant prior to enrollment. The study was conducted in accordance with the principles of the Declaration of Helsinki. All personal information was anonymized, and clinical samples and related data were used strictly for this study.

### 2.2 Inclusion and Exclusion Criteria

Participants were divided into the IC/BPS group and the HC group, with specific inclusion criteria for each group. For the IC/BPS group, participants must meet the diagnostic criteria specified in the Guidelines for Diagnosis and Treatment of Interstitial Cystitis/Bladder Pain Syndrome (2022 Edition). These include the presence of lower urinary tract symptoms (LUTS), daytime frequency (≥ 8 episodes per day), urgency, and chronic suprapubic pain lasting for at least 6 weeks, as well as confirmation of bladder mucosal erythema, petechial hemorrhage, or Hunner's ulcer via cystoscopy. Additional inclusion criteria required: (1) Patients within the age range of 18 to 80 years; (2) No specific IC/BPS treatments (such as bladder instillation or immunosuppressants) within a month before enrollment; (3) Complete analysis of urine sample, urinary ultrasound, and a comprehensive clinical evaluation; and (3) A signed written informed consent. All participants were newly diagnosed and had no previous history of any IC/BPS‐targeted therapies. For the HC group, participants were required to: (1) Have no LUTS or history of urinary system diseases; (2) Be frequency‐matched with the IC/BPS group by age (age difference ≤ 5 years) and sex; (3) Have normal results in urinalysis and urinary ultrasound; (4) Have no systemic diseases that could affect bladder function or urinary biomarkers; and (5) Provide signed written informed consent.

Participants were excluded if they met any of the following criteria: (1) Concurrent urinary tract diseases (e.g., urinary tract infection, bladder stones, bladder cancer, prostate disorders, interstitial nephritis); (2) Systemic diseases (e.g., diabetes mellitus, uncontrolled hypertension, autoimmune diseases, neurological diseases); (3) Use of antibiotics, immunosuppressants, hormones, or anticholinergic drugs within 3 months before enrollment; (4) Pregnancy, lactation, or planned pregnancy during the study period; (5) Presence of psychiatric disorders, cognitive impairment, or inability to cooperate with the study procedures; (6) Fail to provide informed consent; and (7) A history of bladder surgery or urethral injury.

### 2.3 Sample Size Calculation

This three‐class ML study comprised 3 groups: HC, NHIC, and HIC, with 18 predictive features. Sample size was estimated using the Hajian‐Tilaki method for multivariable predictive models. Notably, this calculation accounted for only 18 predictive features and did not adjust for statistical attrition due to screening 127 ML models.

Two thresholds were adopted:

- Ideal criterion: *n*_min_ (ideal) = 10 × *p*
- Minimum acceptable criterion: *n*_min_ (acceptable) = 5 × *p*

where *p* = 18 (number of features).

Accordingly:

- Ideal sample size per group: 10 × 18 = 180
- Minimum acceptable sample size per group: 5 × 18 = 90

A total of 482 eligible participants were enrolled, including 230 HC, 196 NHIC, and 56 HIC subjects. The HC and NHIC groups met the ideal criterion, while the HIC group (a rare subtype, accounting for 11%–15% of IC/BPS cases) was limited by real‐world clinical enrollment constraints and failed to meet the ideal sample‐size benchmark. Multiple small‐sample bias mitigation strategies combined with class weighting (including L1 regularization and coefficient shrinkage) were implemented to address subgroup size imbalance and insufficient statistical power in subtype‐specific prediction.

### 2.4 Clinical Data and Sample Collection

#### 2.4.1 Clinical Data Collection

Trained investigators collected standardized data, including demographic characteristics (age, gender), clinical characteristics [nocturia frequency (NUF), pelvic pain visual analogue scale (VAS)], and urinary ultrasound parameters [functional bladder wall thickness (BWT), functional bladder capacity (FBC)].

All ultrasound scans were conducted by a senior sonographer with at least 5 years of clinical experience. Bladder volume (defined as FBC) was calculated using the formula: 0.52 × transverse diameter × longitudinal diameter × anteroposterior diameter. Functional BWT was measured at the anterior, posterior, and lateral bladder walls; the mean value of three replicate measurements was recorded, with the trigone and vascular structures excluded during measurement.

#### 2.4.2 Urine Sample Collection

Midstream urine was collected into 50 mL sterile enzyme‐free tubes. Specimens were transported on ice at 4°C within 1 h after collection, followed by centrifugation at 1800 r/min for 10 min. The resulting supernatants were stored at −80°C, with freeze–thaw cycles restricted to ≤ 3 times.

#### 2.4.3 Urinary Biomarker Measurement

All quantitative biomarker assays were performed by investigators unaware of group assignments [12, 13]. Before detection, preserved urine supernatants were re‐centrifuged at 12000 r/min for 15 min at 4°C. A Milliplex Human Cytokine/Chemokine Magnetic Bead Panel (Millipore) was utilized to quantify IL‐6, IL‐8, TNF‐α, monocyte chemoattractant protein‐1 (MCP‐1), interferon gamma‐induced protein 10 (IP‐10), macrophage inflammatory protein‐1β (MIP‐1β), regulated upon activation, normal T cell expressed and presumably secreted (RANTES), transforming growth factor‐β (TGF‐β), brain‐derived neurotrophic factor (BDNF), and Eotaxin. TAC was determined by copper ion reduction colorimetric biochemical chromogenic reaction. Additional commercial ELISA kits were used for NGF and oxidative stress biomarkers: nerve growth factor (NGF) was detected using the NGF ELISA Kit (DY256, R&D Systems); 8‐hydroxy‐2′‐deoxyguanosine (8‐OHdG) was measured via the 8‐OHdG ELISA Kit (K4160‐100, Biovision); 8‐iso‐Prostaglandin F2α (8‐iso‐PGF2α) was quantified with the 8‐iso‐PGF2α ELISA Kit (DI‐900‐010, Enzo Life Sciences), and urinary creatinine was detected using the Creatinine Assay Kit (ADI‐907‐030A, Enzo Life Sciences) to correct the concentration of 8‐iso‐PGF2α.

### 2.5 ML Model Construction and Optimization

To develop a diagnostic prediction model for IC/BPS, we stratified and randomly divided the cohort of healthy controls and IC/BPS patients into a training set (*n* = 338) and an external validation cohort (Validation 1, *n* = 144) at a 7:3 ratio. Multi‐dimensional variables encompassing clinical characteristics, urinary ultrasound parameters, and urinary biomarkers were integrated into the ML analytical framework. In total, 127 ML models were established and compared, consisting of traditional regularized regression and ensemble ML algorithms [least absolute shrinkage and selection operator (Lasso), Ridge regression, elastic net (EN), random forest (RF), support vector machine (SVM), generalized linear model boosting (glmBoost), extreme gradient boosting (XGBoost), and other classical algorithms], deep learning (DL) architectures, and partial least squares generalized linear model (plsRglm)‐integrated hybrid regression models. Five‐fold cross‐validation (internal validation) was performed on the training set for hyperparameter tuning to mitigate overfitting risks. Model discriminative performance was primarily quantified using the area under the curve (AUC) of receiver operating characteristic (ROC), a threshold‐independent metric that enables unbiased comparative evaluation across numerous candidate models. We calculated the AUC values of all 127 candidate models on both the training set and Validation 1, and ranked models according to their AUC performance. For extended validation of its generalizability across distinct IC/BPS clinical phenotypes, an additional external cohort (Validation 2) comprising HIC and NHIC patients was introduced. The finalized model derived from healthy controls and overall IC/BPS patients was applied to Validation 2 to test whether model‐derived predictive scores could differentiate HIC from NHIC subgroups, further exploring its potential clinical utility for IC/BPS subtype stratification.

### 2.6 Model Interpretability Analysis (SHAP)

SHAP was used to decompose model predictions into feature contributions, with mean absolute SHAP values indicating feature importance. Summary plots, dependence plots, and force plots were generated for visualization.

### 2.7 Bioinformatics Analysis

#### 2.7.1 Data Sources

Gene expression dataset GSE11783 was downloaded from the Gene Expression Omnibus (GEO, https://www.ncbi.nlm.nih.gov/geo/). Single‐cell RNA‐sequencing (scRNA‐seq) data GSE175526 were also obtained from the GEO database.

#### 2.7.2 GEO Data Processing and DEG Screening

Raw data were preprocessed by the RMA algorithm. Probe annotation was performed using the annotate package in R. Differential expression analysis was conducted using the limma package. DEGs were defined as |log_2_FC| ≥ 1 and *p* < 0.05.

#### 2.7.3 Single Cell Data Analysis

Quality control was performed to remove low‐quality cells, retaining only those with 200–6000 detected genes and < 5% mitochondrial gene content. The Harmony algorithm was applied to correct batch effects. Principal component analysis (PCA) was used for dimensionality reduction, followed by cell clustering using the KNN and Louvain methods. Cell type annotation was conducted using SingleR. Pseudotime trajectory analysis of key cell subsets was performed using Monocle 3. Cell–cell communication networks were constructed and analyzed with CellChat.

#### 2.7.4 Uniform Manifold Approximation and Projection (UMAP)

To visualize cell heterogeneity in low‐dimensional space, UMAP was performed using the top 20 PCA dimensions with the following parameters: n.neighbors = 41, min.dist = 0.35, metric = “euclidean”. UMAP preserved both the local cellular similarity and the global topological structure of single‐cell transcriptome profiles.

#### 2.7.5 Virtual Gene Knockout (scTenifoldKnk)

scTenifoldKnk was used to simulate TNF‐α knockout at the single‐cell level. DEGs were identified and subjected to Gene Ontology (GO) and Kyoto Encyclopedia of Genes and Genomes (KEGG) enrichment using clusterProfiler (*p* < 0.05).

### 2.8 Statistical Analysis

Statistical analyzes were conducted with SPSS 26.0 and R 4.5.2. Normality and homogeneity of variance were assessed using the Shapiro–Wilk test and Levene's test, respectively. Non‐normally distributed data were described as median [Q1, Q3] and analyzed using the Kruskal‐Wallis *H* test or Mann–Whitney *U* test. Non‐normally distributed data across the three groups were first analyzed using a Kruskal‐Wallis test for multiple comparisons. Should the overall differences be significant, Dunn's test, along with Bonferroni correction, was employed to mitigate type I errors in pairwise post hoc comparisons. The Mann–Whitney *U* test was used directly to compare two groups without requiring multiple corrections. Diagnostic performance was evaluated by the AUC, sensitivity, specificity, positive predictive value (PPV), and negative predictive value (NPV). A two‐sided *p* value < 0.05 was considered statistically significant.

## 3 Results

### 3.1 Clinical Indicators and Urinary Biomarker Analysis

#### 3.1.1 Baseline Demographic and Clinical Characteristics and Urinary Ultrasound Parameters Analysis

This study included 482 eligible participants, consisting of 230 HC, 196 NHIC patients, and 56 HIC patients. The age and gender distribution of the three groups did not differ significantly (all *p* > 0.05, Table 1), suggesting favorable comparability of baseline characteristics. Patients in the NHIC and HIC cohorts exhibited significantly more severe clinical manifestations than the HC group, with disease severity becoming more debilitating in the HC group (all *p* < 0.001), with disease severity becoming more debilitating in the HIC group compared to NHIC and HC groups (Figure S1). Clinical characteristics like NUF and pelvic pain visual analogue scale (VAS) score in both the NHIC and HIC groups were statistically significantly elevated. For urinary ultrasound parameters, functional BWT showed significantly increased levels, while FBC was notably decreased in the disease subgroups. Subgroup stratification revealed that the HIC cohort presented with significantly exacerbated clinical symptomatology and more pronounced structural bladder abnormalities, as evidenced by ultrasound measurements, in comparison to the NHIC group. This observation aligns with the established criteria for clinical subtype stratification for IC/BPS.

**TABLE 1:** Baseline demographic and clinical characteristics and urinary ultrasound parameters of study participants.

Variables	HC (*n* = 230)	NHIC (*n* = 196)	HIC (*n* = 56)	*H*	*p*
Gender (M/F, *n*)	11/219	8/188	2/54	1.025	0.358
Age (years)	55.00 (45.00, 71.75)	55.00 (46.75, 68.25)	57.00 (49.75, 63.00)	1.056	0.367
Age Range (years)	38–72	36–70	38–68	—	—
NUF (times)	0 (0, 1)	4 (4, 5)	8 (7, 0)	371.08	< 0.001
Pelvic Pain VAS Score (points)	0 (0, 0)	3 (2, 4)	5 (4, 6)	395.88	< 0.001
Functional BWT (mm)	2.08 (1.67, 2.52)	4.21 (3.57, 4.89)	5.345 (3.93, 6.14)	325.88	< 0.001
FBC (mL)	318.87 (286.00, 333.43)	214.21 (187.26, 256.87)	152.93 (104.68, 179.68)	324.88	< 0.001

*Note:* Data are presented as median [Q1, Q3] for continuous variables and n for categorical variables. Statistical analyzes were performed using the Kruskal‐Wallis *H* test.

#### 3.1.2 Levels of Urinary Biomarkers

Urinary biomarker levels across the HC, NHIC, and HIC groups are summarized in Table 2 and visualized in Figure S1. Pro‐inflammatory cytokines and chemokines showed varied expression patterns between the two groups compared to HC. Most mediators, like TNF‐α, IP‐10, MIP‐1β, and RANTES, were significantly elevated in both NHIC and HIC subgroups compared to HC (all *p* < 0.001). IL‐8 levels were significantly elevated only in the NHIC group (*p* < 0.05), whereas no statistical difference was found between HIC and HC. Conversely, IL‐6 levels were significantly lower in both IC/BPS subtypes compared with HC (all *p* < 0.05). MCP‐1 showed comparable concentrations across all three groups without any intergroup statistical significance. Urinary concentration of TNF‐α (a canonical pro‐inflammatory mediator) was 2.31 times higher in the NHIC subgroup and 2.46 times higher in the HIC subgroup compared to HC (Table 2), highlighting its significant role in the intravesical inflammatory signaling cascade in IC/BPS. Pairwise comparison between NHIC and HIC showed that there were no significant differences in almost all pro‐inflammatory mediators; however, TNF‐α exhibited a stepwise and statistically significant increase from NHIC to HIC (*p* < 0.05). This result aligns to a degree with the observed patterns in clinical characteristics and supports a graded correlation between specific urinary mediators and IC/BPS disease severity.

**TABLE 2:** Levels of urinary biomarkers in HC, NHIC, and HIC groups.

Urinary biomarkers	HC (*n* = 230)	NHIC (*n* = 196)	HIC (*n* = 56)	*H*	*p*
Pro‐inflammatory Cytokines/Chemokines					
IL‐6 (pg/mL)	0.88 (0.65, 1.23)	0.55 (0.28, 1.44)	0.45 (0.26, 0.69)	28.75	< 0.001
IL‐8 (ng/mL)	6.77 (1.42, 19.31)	9.18 (1.97, 26.01)	11.26 (1.85, 28.27)	9.89	< 0.001
IP‐10 (pg/mL)	4.11 (1.89, 33.38)	1.82 (1.28, 4.48)	1.77 (1.27, 4.77)	57.32	< 0.001
MCP‐1 (pg/mL)	162.32 (66.48, 271.06)	158.98 (66.08, 286.45)	154.39 (66.15, 279.60)	1.07	0.3
TNF‐α (pg/mL)	0.71 (0.63, 0.92)	1.64 (1.42, 1.73)	1.75 (1.57, 1.93)	184.36	< 0.001
MIP‐1β (pg/mL)	1.61 (1.18, 3.50)	0.29 (0.18, 1.38)	0.29 (0.16, 1.21)	129.15	< 0.001
RANTES (pg/mL)	4.91 (2.63, 8.20)	1.64 (0.64, 4.44)	1.30 (0.58, 3.58)	92.30	< 0.001
Oxidative Stress Biomarkers					
8‐iso‐PGF2α (ng/mg Cr)	13.22 (7.40, 21.48)	27.73 (10.22, 58.23)	38.71 (15.55, 62.26)	81.98	< 0.001
8‐OHdG (ng/mL)	15.40 (7.02, 30.89)	21.94 (12.94, 41.02)	27.01 (14.63, 54.26)	26.28	< 0.001
Antioxidant Indicators					
TAC (U/mL)	724.62 (511.48, 1638.41)	718.30 (382.55, 1267.69)	714.45 (312.46, 1162.46)	11.52	< 0.001
Other Biomarkers					
TGF‐β (ng/mL)	6.67 (4.79, 8.25)	11.38 (5.15, 14.64)	15.30 (5.26, 18.58)	84.22	< 0.001
BDNF (ng/mL)	0.54 (0.44, 0.68)	0.53 (0.43, 0.66)	0.51 (0.42, 0.62)	0.60	0.44
Eotoxin (pg/mL)	3.84 (2.52, 6.78)	2.94 (2.58, 5.21)	2.43 (2.10, 5.21)	13.51	< 0.001
NGF (pg/mL)	0.24 (0.20, 0.26)	0.16 (0.14, 0.21)	0.15 (0.13, 0.19)	123.90	< 0.001

*Note:* Data are presented as median [Q1, Q3]. Statistical analyzes were performed using the Kruskal‐Wallis *H* test.

Biomarkers of oxidative stress (8‐OHdG and 8‐iso‐PGF2α) were substantially upregulated in both IC/BPS subtypes relative to HC, accompanied by a compensatory reduction in the antioxidant indicator TAC, which reflects disrupted redox homeostasis within the bladder microenvironment (all *p* < 0.05). No significant differences in these oxidative stress and antioxidant indices were identified between the NHIC and HIC subgroups.

The analysis of other miscellaneous biomarkers showed no notable intergroup differences in BDNF between HC, NHIC, and HIC. TGF‐β levels increased progressively with increasing disease severity (*p* < 0.01). Eotaxin and NGF were significantly different particularly between HC and NHIC (all *p* < 0.01), with no significant differences observed between the two IC/BPS subtypes.

### 3.2 ML Model Construction and Diagnostic Performance Analysis

#### 3.2.1 Diagnostic Efficacy of Single Indicators

**FIGURE 1:**
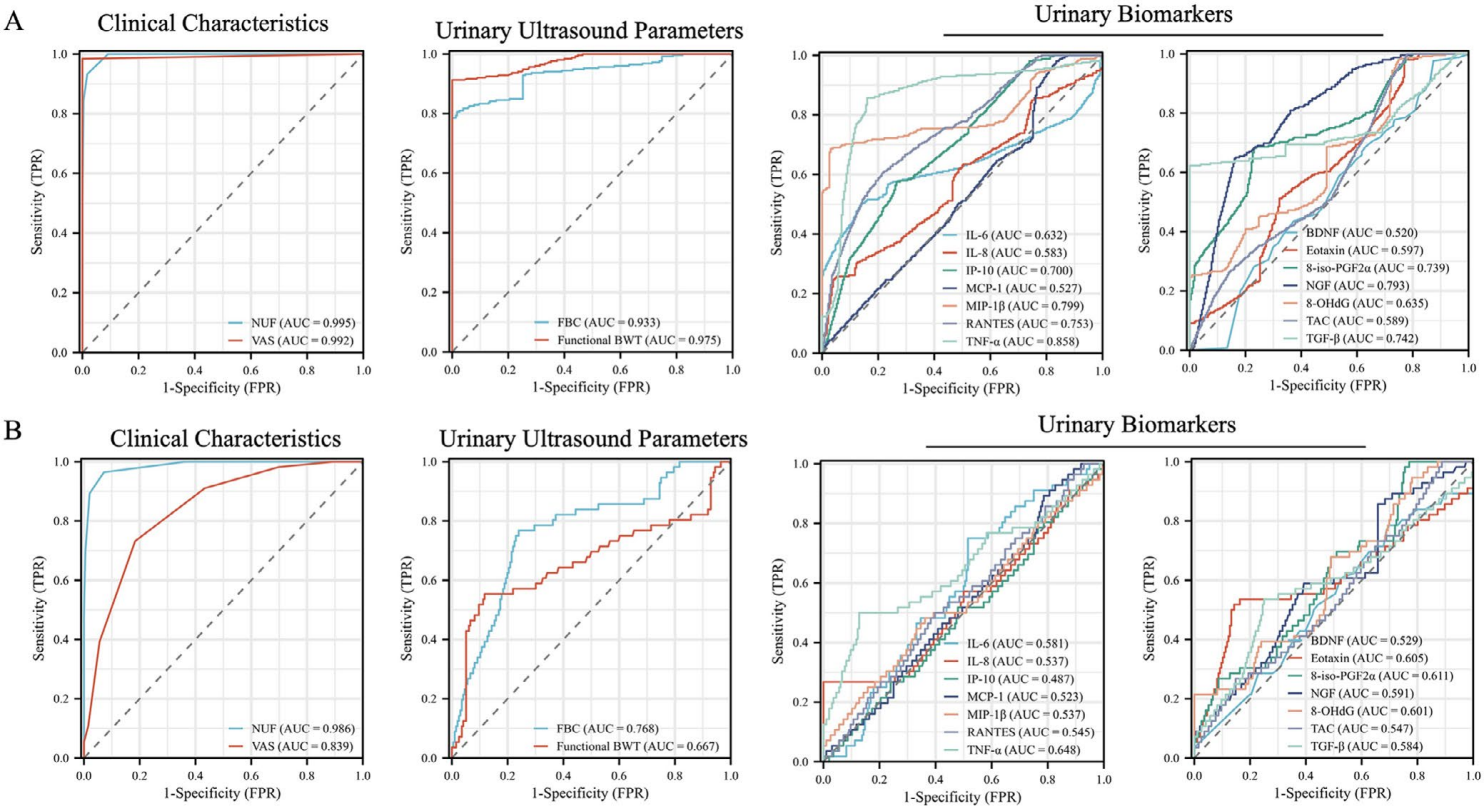
Diagnostic performance of clinical, urinary, and oxidative stress markers for IC/BPS and its subtypes. (A) ROC curves for evaluating the discriminatory capacity of clinical characteristics, urinary ultrasound parameters, and urinary biomarkers between HC and IC/BPS patients. (B) ROC curves for evaluating the discriminatory capacity of clinical characteristics, urinary ultrasound parameters, and urinary biomarkers between NHIC and HIC patients. The diagonal dashed line indicates a random classifier with an AUC of 0.5. Corresponding AUC values for each indicator are listed in the plot legends.

To evaluate the diagnostic value of individual clinical and urinary biomarkers for IC/BPS and its subtypes, ROC curve analysis was performed. As shown in Figure 1A and Table S1, clinical characteristics and urinary ultrasound parameters exhibited the highest diagnostic efficacy in differentiating IC/BPS patients from HC. NUF achieved the highest AUC of 0.995, followed closely by VAS with an AUC of 0.992, functional BWT at 0.975, and FBC with an AUC of 0.933. TNF‐α demonstrated the best diagnostic efficacy (AUC = 0.858) among urinary biomarkers. ROC analysis for subtype discrimination between NHIC and HIC is shown in Table S2 and Figure 1B. Clinical characteristics remained the most reliable differentiators, with NUF achieving an AUC of 0.986, followed by VAS (AUC = 0.839). With AUCs ranging from 0.487 to 0.648, urinary biomarkers performed poorly in differentiating IC/BPS subtypes. However, TNF‐α had the highest AUC of 0.648 among these biomarkers, which reflects only a limited ability to differentiate between NHIC and HIC subtypes. However, these findings do not undermine the established role of TNF‐α as a vital inflammatory mediator involved in the progression of the disease.

#### 3.2.2 Construction and Optimization of ML Models

**FIGURE 2:**
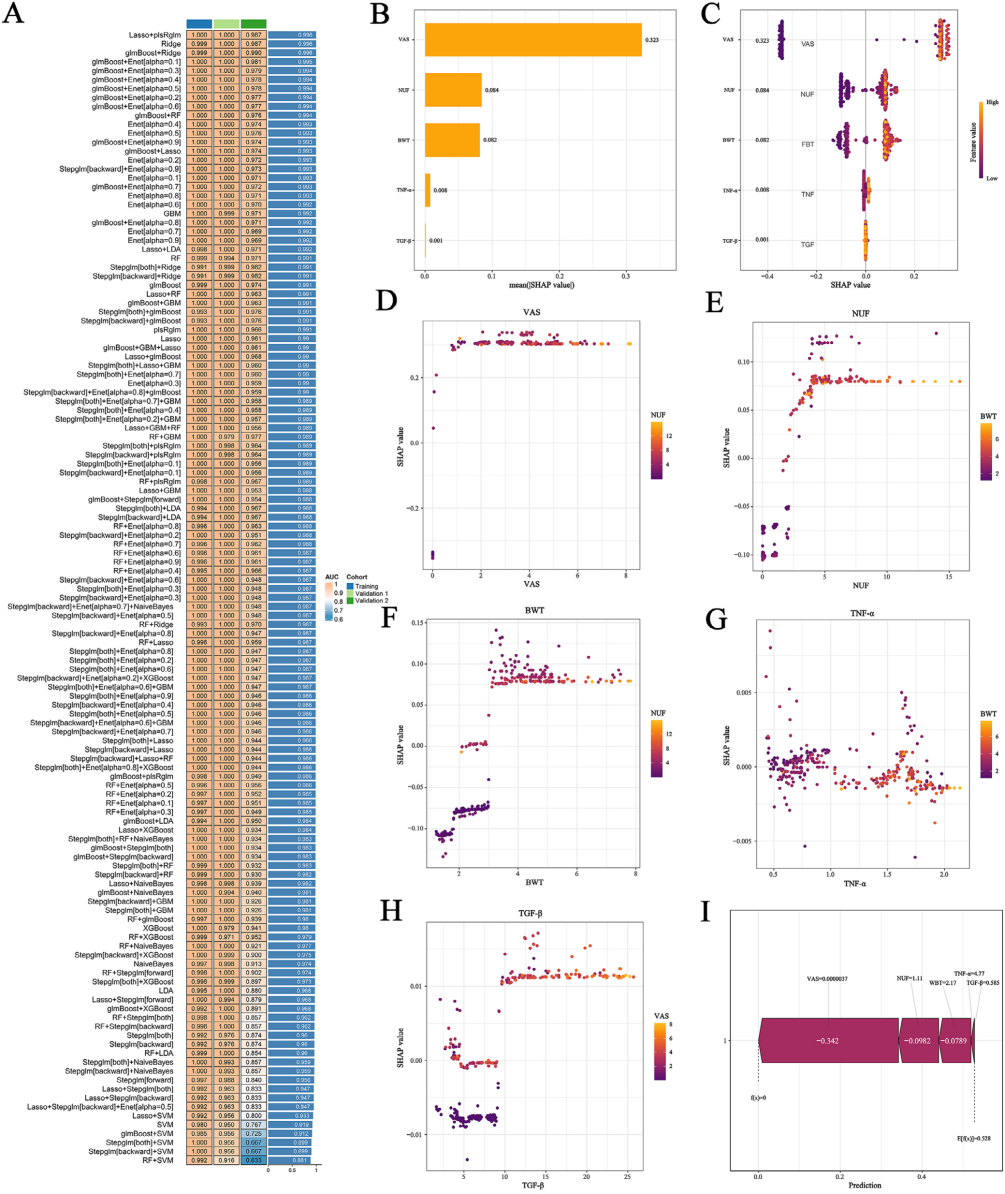
Construction and external validation of a diagnostic prediction model for IC/BPS using multi‐dimensional indicators. (A) Heatmap of 127 models selected by Lasso and stepwise regression. Color intensity corresponds to each feature's relative predictive importance or expression level, with sample cohorts (Validation 1 and Validation 2) annotated at the top. (B) Bar chart of mean SHAP values for the top predictive features, quantifying the contribution of each feature to the model. (C) Violin plots of SHAP value distributions for all key features, demonstrating their directional impacts on model predictions. (D–H) Scatter plots displaying the distribution patterns of core clinical indicators. (I) SHAP force plot illustrating the additive contribution of each feature.

A total of 127 ML models were constructed, covering traditional ML, DL, and other algorithms, to develop an optimal diagnostic model for IC/BPS. As shown in Figure 2A and Table S3, the performance of each model was systematically evaluated. Most algorithms reached an AUC of 1.000 in both training and validation cohorts, showing nearly perfect discriminative capacity. Among all models, the Lasso+plsRglm algorithm maintained stable predictive performance across all validation datasets and exhibited satisfactory generalization ability. After comprehensive evaluation integrating discriminative efficacy, feature screening function, collinearity correction, and clinical interpretability, Lasso+plsRglm was selected as the final optimal diagnostic model.

#### 3.2.3 Model Interpretability and Key Feature Identification via SHAP Analysis

To clarify the contribution of each feature to model prediction, SHAP analysis was performed. As shown in Figure 2B, the mean SHAP value plot indicated that NUF, functional BWT, and VAS score were the three most important features, followed by TNF‐α, which represented the most critical urinary biomarker. The SHAP summary plot illustrates the direction and magnitude of each feature's influence on model prediction (Figure 2C). Consistent with the clinical and molecular features of IC/BPS, higher levels of NUF, VAS score, functional BWT, and TNF‐α were correlated with a higher likelihood of IC/BPS diagnosis. Dependence plots for the key features (Figure 2D–H) further confirmed their dose–response relationships with model prediction. For example, Figure 2G showed a strong positive association between TNF‐α levels and SHAP values, which indicated that higher TNF‐α concentrations significantly increased the likelihood of IC/BPS diagnosis. Lastly, Figure 2I showed the model's predictive performance. Box plots showed that the HC group, NHIC group, and HIC group had significantly different predicted probabilities, confirming the model's capacity to differentiate between disease severity.

### 3.3 Transcriptomic Analysis Based on GEO Datasets

#### 3.3.1 Data Preprocessing and Sample Quality Control

Transcriptome analysis was used on the public dataset GSE11783 from the GEO database to systematically investigate the molecular pathways causing IC/BPS. PCA was conducted to assess data quality and sample separation (Figure S2A). Figure S2B clearly demonstrated distinct clustering differences between HC subjects and patients with IC/BPS, as well as between NHIC and HIC groups, with clear separation in the principal component space. This robust separation confirmed the quality of the preprocessed data and intrinsic transcriptional differences among distinct disease statuses, laying a solid foundation for subsequent differential expression analysis.

#### 3.3.2 Identification of DEGs

**FIGURE 3:**
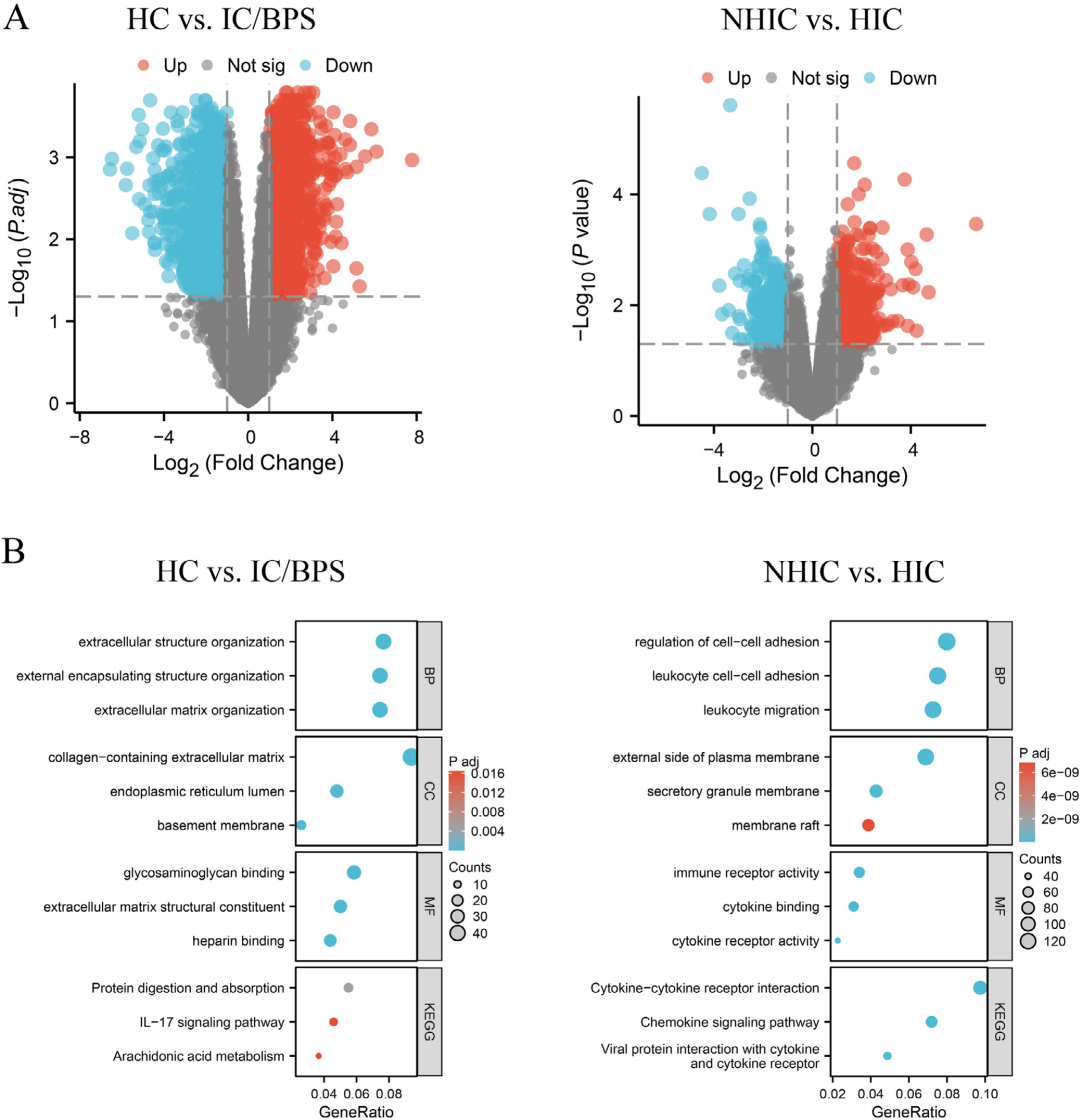
Transcriptomic profiling and functional enrichment analysis in IC/BPS and its subtypes. (A) Volcano plots showing DEGs in HC versus IC/BPS (left) and NHIC versus HIC (right) cohorts. The X‐axis shows log_2_‐transformed fold change (log_2_FC), and the Y‐axis shows −log_10_‐transformed adjusted *p*‐values. Red and blue dots represent significantly upregulated and downregulated genes, respectively; gray dots indicate non‐significant genes. Dashed lines denote the significance thresholds (|log_2_FC| ≥ 1, adjusted *p* < 0.05). (B) Functional enrichment analysis of DEGs, including GO terms and KEGG pathways.

To find transcriptional signatures connected to IC/BPS and its subtypes, differential expression analysis was employed. Volcano plots were generated to display DEGs with a threshold set at |log_2_FC| ≥ 1 and *p* < 0.05 (Figure 3A). NHIC, compared to HIC (right panel, Figure 3A), showed more notable transcriptional divergence with a higher number of significantly changed genes, thereby revealing significant molecular heterogeneity between these two clinical phenotypes. To further confirm the DEG expression patterns, box plots and heatmaps were constructed. Figure S2A showed consistent upregulation of key signature DEGs in IC/BPS patients compared with HC and in HIC compared with NHIC. A heatmap of the top DEGs was shown in Figure S2C, which displayed sample clustering stratified by group. Tescalcin (TESC) and cartilage acidic protein 1 (CRTAC1) were consistently downregulated in the IC/BPS cohort, while neutrophil cytosolic factor 2 (NCF2) and interleukin 3 receptor subunit alpha (IL3RA) were markedly upregulated, as visualized in the heatmap. Additionally, distinct transcriptional profiles between the NHIC and HIC subgroups were observed, as expressed by marker genes such as SH3 domain containing GRB2 like, endophilin B2 (SH3GLB2) and bicaudal C homolog 1 (BICC1). These observations summarize the expression signatures specific to subgroups and confirm the reliability of the outcomes from DEG screening.

#### 3.3.3 Functional Enrichment Analysis

GO and KEGG enrichment analyzes of identified DEGs were performed to highlight the biological functions and key signaling pathways implicated in the pathophysiology of IC/BPS. The most significantly enriched GO terms and KEGG pathways are depicted in Figure 3B. Extracellular structure organization and external encapsulating structure organization were found to be the most significantly enriched biological processes (BPs) between HC and IC/BPS patients, which suggests extensive remodeling of the bladder extracellular matrix (ECM) in IC/BPS. Molecular functions (MFs) analysis identified heparin and glycosaminoglycan binding, indicating bladder mucosal barrier disruption. In addition, arachidonic acid metabolism and the IL‐17 signaling pathway were significantly enriched. This highlights the crucial participation of immunological inflammation and lipid mediator metabolism in the advancement of disease. In the comparison between NHIC and HIC, functional enrichment revealed cell–cell adhesion and leukocyte migration as the most prominent BPs (Figure 3B). Membrane‐related terms, including the external side of the plasma membrane and secretory granule membrane, were significantly enriched. The cytokine‐cytokine receptor interaction and chemokine signaling pathway were consistently found to be the most dysregulated pathways by KEGG pathway analysis, further validating the significance of inflammatory mediator signaling and immune cell recruitment in the formation of ulcers in HIC.

### 3.4 Single‐Cell Transcriptomic Analysis Reveals Distinct Inflammatory Cellular States in IC/BPS

**FIGURE 4:**
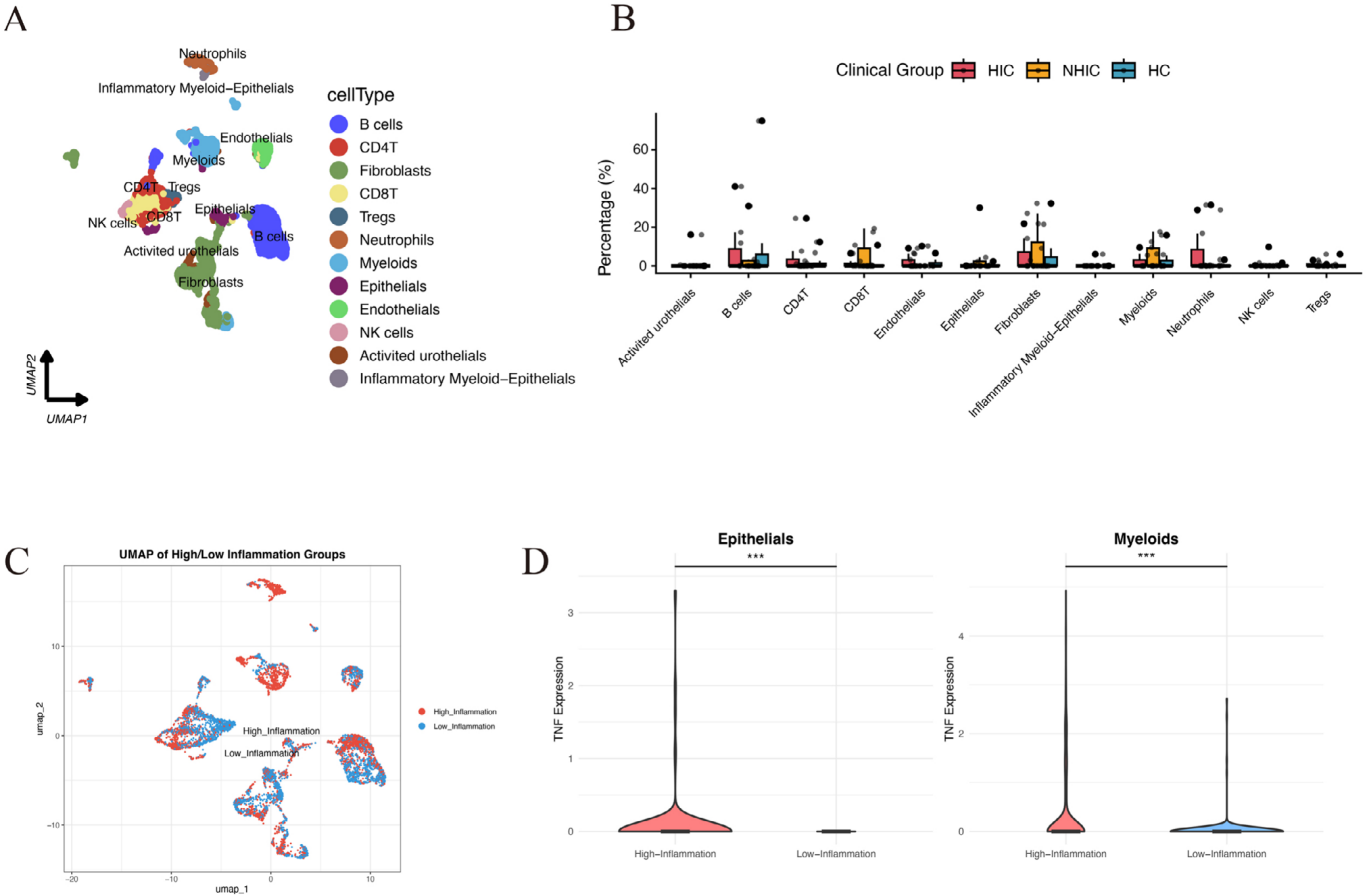
Single‐cell transcriptomic profiling of bladder tissues identifies distinct inflammatory cell states in IC/BPS. (A) UMAP visualization of bladder scRNA‐seq data, color‐annotated by 12 distinct cell types, including immune cells, stromal cells, and epithelial subsets. (B) Cell type composition analysis across three clinical groups: HIC (red), NHIC (yellow), and HC (blue), revealing differential immune cell infiltration between IC/BPS subtypes. (C) UMAP projection of all cells stratified into high‐inflammation subgroup (red) and low‐inflammation subgroup (blue) based on inflammatory gene expression signatures. (D) Violin plots showing significantly elevated *TNF* expression in epithelial and myeloid cells derived from the high‐inflammation subgroup.

To dissect the cellular heterogeneity and inflammatory status of IC/BPS, we performed scRNA‐seq on bladder tissue samples from patients with HIC, NHIC, and HC. Following stringent quality control (Figure S3A,B), unsupervized clustering analysis identified 12 distinct cell populations, encompassing immune cells [B cells, CD4^+^ T cells, CD8^+^ T cells, regulatory T cells (Tregs), natural killer (NK) cells, neutrophils, and myeloid cells], stromal cells (fibroblasts and endothelial cells), and epithelial cells (epithelial cells, activated urothelial cells, and inflammatory myeloid‐epithelial cells) (Figures 4A and S3C,D).

Cell composition analysis revealed significant differences in immune cell infiltration across clinical groups (Figures 4B and S3E). An inflammation score was calculated for each cell based on the expression of a curated inflammatory gene profile to further stratify cellular inflammatory states. This distinguished two functionally distinct cell states: high‐inflammatory and low‐inflammatory (Figure 4C). TNF expression and inflammation score were found to be significantly positively correlated across various cell lineages, with the strongest correlations found in myeloid cells (*r* = 0.484, *p* < 0.001) and epithelial cells (*r* = 0.443, *p* < 0.001) (Figure 4D).

**FIGURE 5:**
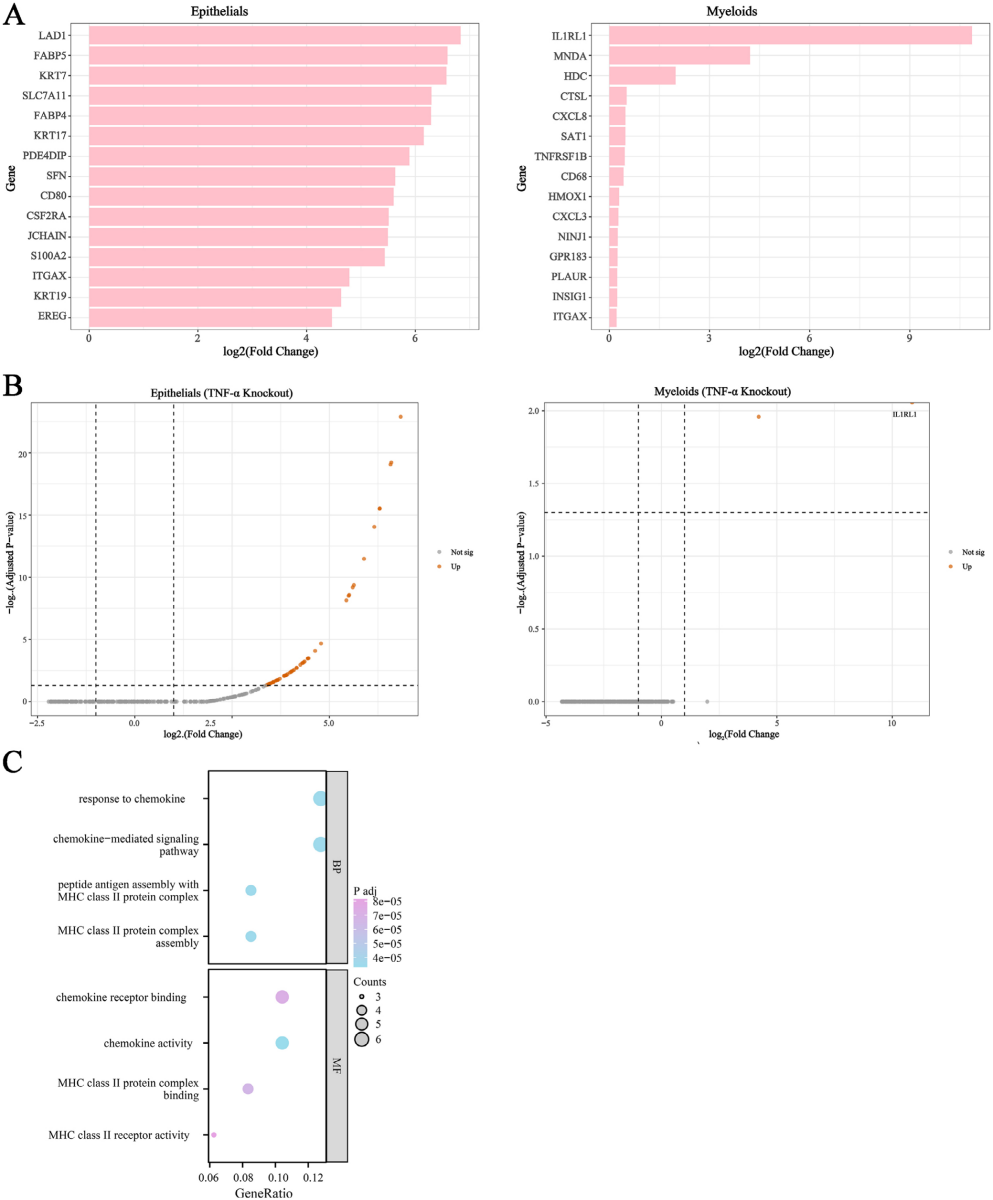
Virtual gene knockout validates the pro‐inflammatory function of TNF in epithelial and myeloid cells of IC/BPS. (A) Heatmaps depicting transcriptomic alterations in epithelial and myeloid cells following virtual TNF knockout, highlighting the downregulation of pro‐inflammatory gene programs. (B) Volcano plots of DEGs after virtual TNF knockout in epithelial and myeloid cells, quantifying the degree of inflammatory gene suppression. (C) GO enrichment analysis of dysregulated genes following epithelial TNF knockout, demonstrating significant attenuation of chemokine‐mediated signaling (BP) and immune receptor activity (MF) pathways.

Differential gene expression analysis between high‐ and low‐inflammatory cells identified 286 upregulated genes and 194 downregulated genes (Figure S3F,G). Functional enrichment analysis demonstrated that upregulated genes were significantly enriched in inflammatory response pathways, including cytokine‐cytokine receptor interaction, chemokine signaling, and leukocyte migration (Figure S3H). In the high‐inflammation subgroup, cell–cell communication analysis revealed a much more enhanced inflammatory signaling network with more contacts between T cells, myeloid cells, and epithelial cells (Figure S3I,J). Pseudotime trajectory analysis confirmed a continuous transition of cellular states from low‐inflammation to high‐inflammation phenotypes, accompanied by dynamic upregulation of core inflammatory cytokines (CCL2, IL1B, IL6, TNF, CXCL8) along the trajectory (Figure S3K–M). To determine the key functional role of TNF in the inflammatory IC/BPS milieu, independent in silico TNF‐α knockout simulations using the scTenifoldKnk algorithm in epithelial and myeloid cells were performed (Figure S3N). Silencing of TNF induced significant transcriptomic alterations in both cell types, as illustrated in Figure 5A. Key proinflammatory cytokines and chemokines, including IL1B, IL6, CCL2, and CXCL8, were significantly downregulated. TNF suppression significantly reduced proinflammatory transcriptional programs in both the epithelial and myeloid compartments, as confirmed by volcano plots (Figure 5B). Subsequently, GO enrichment analysis was conducted on the dysregulated genes in epithelial cells induced by TNF knockout (Figure 5C). BP terms related to inflammatory responses, chemokine‐mediated signaling, and leukocyte migration were significantly enriched in the dysregulated genes. In addition, MF terms were predominantly concentrated in chemokine activity, cytokine receptor binding, and immune receptor activity. All of these findings suggest that TNF plays a crucial role in maintaining the hyperinflammatory state in IC/BPS. In silico TNF‐α knockout was performed to predict its potential impact as a pro‐inflammatory factor in this condition and revealed its significance in this disease as a key inflammatory marker and as an exploitable target for diagnosis and potential therapy. These findings are based on computational inference and will require experimental validation in large cohorts to confirm their biological relevance.

## 4 Discussion

This study conducted a comprehensive, multidimensional investigation of IC/BPS, focusing on diagnostic biomarker screening, molecular mechanism analysis, and subtype classification. Using clinical characteristics, urinary ultrasound parameters, urinary biomarkers, ML modeling, public transcriptome data mining, and scRNA‐seq, we systematically identified the core inflammatory regulatory network and cellular heterogeneity of IC/BPS. This work provides evidence‐based support and theoretical references for the accurate diagnosis, subtype differentiation, and targeted therapy of this disease.

We examined available transcriptome data from the GEO database to investigate the inflammatory features of IC/BPS. The results showed that TESC and CRTAC1 were consistently downregulated in IC/BPS, while NCF2 and IL3RA were significantly upregulated. The genes identified may be used as molecular indicators to differentiate normal bladder tissue from IC/BPS. Furthermore, DEGs, such as SH3GLB2 and BICC1, offer possible targets for IC/BPS subtype classification (HIC and NHIC). Pathway enrichment analysis revealed that DEGs between IC/BPS and HC were mainly enriched in inflammation‐related pathways, accompanied by enhanced immune cell adhesion. These findings confirm that abnormal activation of inflammatory factors and immune cell infiltration are key drivers of IC/BPS pathogenesis, which is consistent with key inflammatory biomarkers identified by ML models [14].

Further transcriptome comparison between HIC and NHIC groups demonstrated that their DEGs were primarily enriched in extracellular matrix remodeling and metabolic reprogramming pathways. This suggests that IC/BPS subtype classification should integrate both inflammatory status and tissue remodeling features [12, 15, 16]. Measurement results showed that key pro‐inflammatory mediators such as TGF‐β1, CCL5, and TNF‐α were significantly elevated in IC/BPS patients. Moreover, chemokine expression was higher in the HIC group than in the NHIC group, indicating a positive correlation between inflammatory intensity and disease severity. This provides a theoretical basis for anti‐inflammatory treatment.

To address cellular heterogeneity that cannot be resolved by conventional bulk sequencing, we performed scRNA‐seq analysis. The results revealed obvious remodeling of the cellular microenvironment in IC/BPS patients, especially in the HIC group. The proportion of normal urothelial cells decreased, while that of activated urothelial cells increased, indicating impaired urothelial barrier function. This represents a key cellular basis for bladder mucosal injury and increased permeability in patients. The cellular profile of the NHIC group presented an intermediate phenotype between HIC and HC, characterized by moderate immune infiltration and epithelial abnormalities. These results are consistent with gene expression profiling and provide cytological support for the clinical classification of IC/BPS [17, 18].

Significant differences in molecular expression and cell–cell communication were observed between the high‐ and low‐inflammation subgroups. DEGs were mainly enriched in immune‐related processes such as T cell differentiation and monocyte differentiation. The high‐inflammation subgroup exhibited more abundant and stronger intercellular interactions. A significant, pseudotime‐dependent increase in *CCL2* and *IL6* expression was observed, indicating that inflammatory status is tightly coupled with cellular differentiation and that dynamic inflammatory gene expression is critical for the pathological phenotype of IC/BPS [19, 20, 21]. Cell–cell communication and pseudotime analyzes further delineated the regulatory framework of the inflammatory microenvironment in IC/BPS. In the high‐inflammation subgroup, neutrophils, myeloid cells, and epithelial cells formed an intensive signaling network centered on the TNF pathway, which mediates crosstalk between inflammatory and target cells and represents a key mechanism driving the hyperinflammatory state [22]. Pseudotime analysis revealed for the first time the dynamic differentiation trajectory of epithelial cells under inflammatory conditions: from a quiescent state to an activated state and then to an inflammatory hyperplastic state. Differentiation nodes of urothelial cells and urothelial cells were closely adjacent, suggesting that immune‐epithelial crosstalk is a core driver of urothelial cell activation and cell fate determination. This finding provides new insights into the mechanisms of epithelial injury and targeted therapy for urothelial cell transdifferentiation in IC/BPS [23].

Using scTenifoldKnk, we simulated TNF‐α knockdown [24] to predict the role of TNF‐α in regulating the epithelial‐myeloid inflammatory axis in IC/BPS. Our results demonstrated that TNF knockout significantly reduced the expression of multiple downstream pro‐inflammatory mediators. Silencing TNF in epithelial cells inhibited IC/BPS core pathogenic pathways, including immune cell recruitment and chemokine signaling.

In summary, this study established a comprehensive framework involving clinical phenotypes, non‐invasive diagnostic models, molecular mechanisms, and cellular targets. We identified the TNF‐α‐mediated inflammatory‐oxidative stress loop and abnormal immune‐epithelial crosstalk as core pathogenic mechanisms of IC/BPS, with activated urothelial cells defined as key therapeutic targets. An interpretable and universal ML diagnostic model was also constructed. These findings provide cytological support for IC/BPS clinical classification and novel theoretical and translational evidence for precise subtyping, early diagnosis, and targeted therapy, with important scientific and clinical significance.

This study has several limitations. First, this study is limited by its single‐center retrospective design for initial model development and validation. While model performance was verified using two independent external validation cohorts, future multi‐center prospective studies are warranted to fully establish its clinical generalizability. Second, the regulatory role of TNF‐α is supported only by bioinformatic analysis and virtual gene knockout, lacking in vitro and in vivo experimental verification. Third, no long‐term follow‐up data are available to evaluate the prognostic value of key biomarkers. Fourth, the Hajian‐Tilaki sample size estimation accounted only for 18 predictive features, neglecting the statistical penalties introduced by screening 127 ML models. Massive model comparisons inflate the effective degrees of freedom and exacerbate overfitting risks. While 5‐fold cross‐validation was utilized to partially counteract this bias, potential residual confounding remains an unaddressed risk. Future research may adopt sample‐size formulas accounting for multiple model comparisons to further constrain overfitting. Additionally, the rare HIC subtype (*n* = 56) did not reach the ideal Hajian‐Tilaki sample threshold due to clinical enrollment restrictions. Though class weighting, regularization, and coefficient shrinkage reduced small‐sample bias, subtype‐specific prediction conclusions remain preliminary and need validation in expanded independent HIC cohorts. Finally, the results were obtained from a Han Chinese population and require further validation in other ethnic groups.

## 5 Conclusion

IC/BPS shows significant heterogeneity in clinical manifestations, molecular characteristics, and cellular features, among which HIC is a more severe inflammatory subtype. The ML model established based on clinical characteristics, urinary ultrasound parameters, and urinary biomarkers allows for high‐precision, non‐invasive, and stratified diagnosis of this disease. The core pathogenesis of IC/BPS includes damage to the urothelial barrier, remodeling of the extracellular matrix, abnormal infiltration of immune cells, and persistent overactivation of inflammatory signaling pathways. Single‐cell transcriptomic analysis further confirms that the epithelial‐myeloid/monocytic crosstalk axis is the core functional unit that drives the formation and maintenance of the highly inflammatory state in bladder tissue. As a key hub molecule, TNF‐α participates prominently in inflammatory amplification loops, mucosal injury, and disease progression. It exhibits excellent potential as a reliable non‐invasive biomarker to distinguish IC/BPS from HC, as well as a promising therapeutic target.

## Author Contributions

**Cheng Luo:** conceptualization, methodology, formal analysis, data curation, writing − original draft, writing − review and editing. **Yonghui Guan:** data curation, writing − review and editing, project administration. **Yifala Dilimulati:** formal analysis, data curation, visualization. **Xinping Zhang:** investigation, data curation. **Cancan Wang:** data curation, writing − original draft. **Mulati Rexiati:** conceptualization, supervision, funding acquisition.

## Funding

This work was supported by the Regional Science Fund of the National Natural Science Foundation of China (Grant No. 82260139), and the Health Commission of Xinjiang Uygur Autonomous Region under the Youth Medical Science and Technology Talent Special Research Project (Grant WJWY‐202312).

## Conflicts of Interest

The authors declare no conflicts of interest.

## Supporting information


**Figure S1:** Differences in clinical indicators (NUF, VAS), urinary ultrasound parameters (FBC, functional BWT), and urinary biomarkers (IL‐6, IL‐8, TNF‐α, MCP‐1, IP‐10, MIP‐1β, RANTES, 8‐OHdG, 8‐iso‐PGF2α, TAC, BDNF, TGF‐β, Eotaxin, NGF) among HC, NHIC, and HIC groups. ^ns^ *p* > 0.05, *p < 0.05, **p < 0.01, ***p < 0.005, ****p < 0.001.

**Figure S2:** Transcriptomic analysis identifies distinct gene expression signatures in IC/BPS patients compared with HC. (A) Normalized signal intensities of differentially expressed probes in HC versus IC/BPS and NHIC versus HIC cohorts, demonstrating consistent upregulation. (B) PCA plots demonstrate clear separation between HC and IC/BPS patient groups, with PC1 and PC2 explaining the majority of the total variance. (C) Unsupervized hierarchical clustering of DEGs reveals distinct expression patterns in HC versus IC/BPS and NHIC versus HIC cohorts. Color scale indicates z‐score‐normalized expression levels: blue indicates downregulation and red indicates upregulation.

**Figure S3:** Comprehensive single‐cell analysis of bladder tissue in IC/BPS, indicating quality control, cell annotation, inflammation stratification, and cell‐cell communication. (A) scRNA‐seq quality control to ensure data reliability. (B) Highly variable gene selection. (C) UMAP cell clustering. (D) Marker gene expression for cell type annotation. (E) Cell type composition across clinical groups. (F) Volcano plot of DEGs between high‐ and low‐inflammation subgroups. (G) Heatmap of top DEGs. (H) Functional enrichment analysis of DEGs. (I, J) Cell‐cell interaction networks in low‐ and high‐inflammation subgroups, showing enhanced inflammatory signaling in the high‐inflammation group. (K, L) Pseudotime trajectory analysis revealing a continuous transition from low‐ to high‐inflammation cell states. (M) Dynamic expression of core inflammatory cytokines along pseudotime. (N) Expression and distribution of TNF‐α in different cell types.

**Figure S4:** Correlation between inflammation score and TNF expression across bladder cell types in IC/BPS. Scatter plots demonstrating the positive correlation between inflammation score and TNF expression in multiple bladder cell populations, including Epithelials, Myeloids, CD4^+^ T cells, NK cells, CD8^+^ T cells, Tregs, Fibroblasts, Inflammatory Myeloid‐Epithelials, Activated urothelia, B cells, Endothelials, and Neutrophils. Linear regression lines and 95% confidence intervals (Cls) are shown, with correlation coefficients (*r*) and *p*‐values indicating statistical significance.

**Table S1:** ROC analysis results of single indicators for distinguishing IC/BPS from HC.

**Table S2:** ROC analysis results of single indicators for distinguishing NHIC from HIC.

**Table S3:** Features screened by each ML algorithm.

## Data Availability

The original contributions presented in the study are included in the article/Supporting Information. Further inquiries can be directed to the corresponding authors.
