# Supplementary material for: Diagnostic Model Development for IC/BPS and Its Subtypes Using Clinical Indicators, Urinary Biomarkers, and Single‐Cell Transcriptomic Analysis
Source: FASEB J. 2026 Aug 4;40(15):e72173. doi: 10.1096/fj.202602743R (PMC13435336; doi:10.1096/fj.202602743R)

Figure S1 Differences in clinical indicators (NUF, VAS), urinary ultrasound parameters (FBC, functional BWT), and urinary biomarkers (IL-6, IL-8, TNF-α, MCP-1, IP-10, MIP-1β, RANTES, 8-OHdG, 8-iso-PGF2α, TAC, BDNF, TGF-β, Eotaxin, NGF) among HC, NHIC, and HIC groups. $^{ns}P>0.05$, $*P<0.05$, $**P<0.01$, $***P<0.005$, $****P<0.001$.

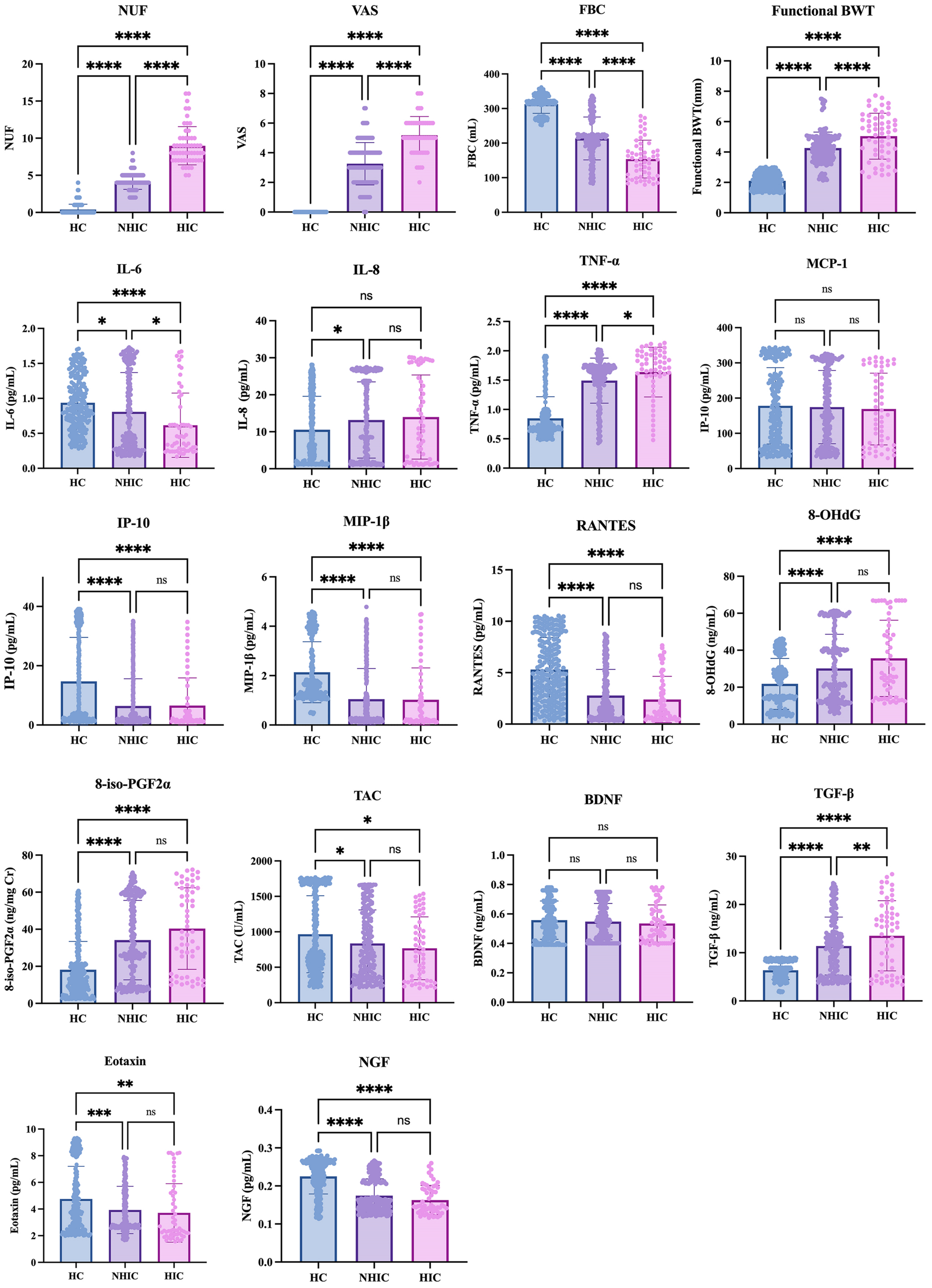

Supplement: Supplementary file 1 [file FSB2-40-e72173-s003.pdf]
