# Supplementary material for: Diagnostic Model Development for IC/BPS and Its Subtypes Using Clinical Indicators, Urinary Biomarkers, and Single‐Cell Transcriptomic Analysis
Source: FASEB J. 2026 Aug 4;40(15):e72173. doi: 10.1096/fj.202602743R (PMC13435336; doi:10.1096/fj.202602743R)

Figure S3 Comprehensive single-cell analysis of bladder tissue in IC/BPS, indicating quality control, cell annotation, inflammation stratification, and cell-cell communication. (A) scRNA-seq quality control to ensure data reliability. (B) Highly variable gene selection. (C) UMAP cell clustering. (D) Marker gene expression for cell type annotation. (E) Cell type composition across clinical groups. (F) Volcano plot of DEGs between high- and low-inflammation subgroups. (G) Heatmap of top DEGs. (H) Functional enrichment analysis of DEGs. (I-J) Cell-cell interaction networks in low- and high-inflammation subgroups, showing enhanced inflammatory signaling in the high-inflammation group. (K-L) Pseudotime trajectory analysis revealing a continuous transition from low- to high-inflammation cell states. (M) Dynamic expression of core inflammatory cytokines along pseudotime. (N) Expression and distribution of TNF-α in different cell types.

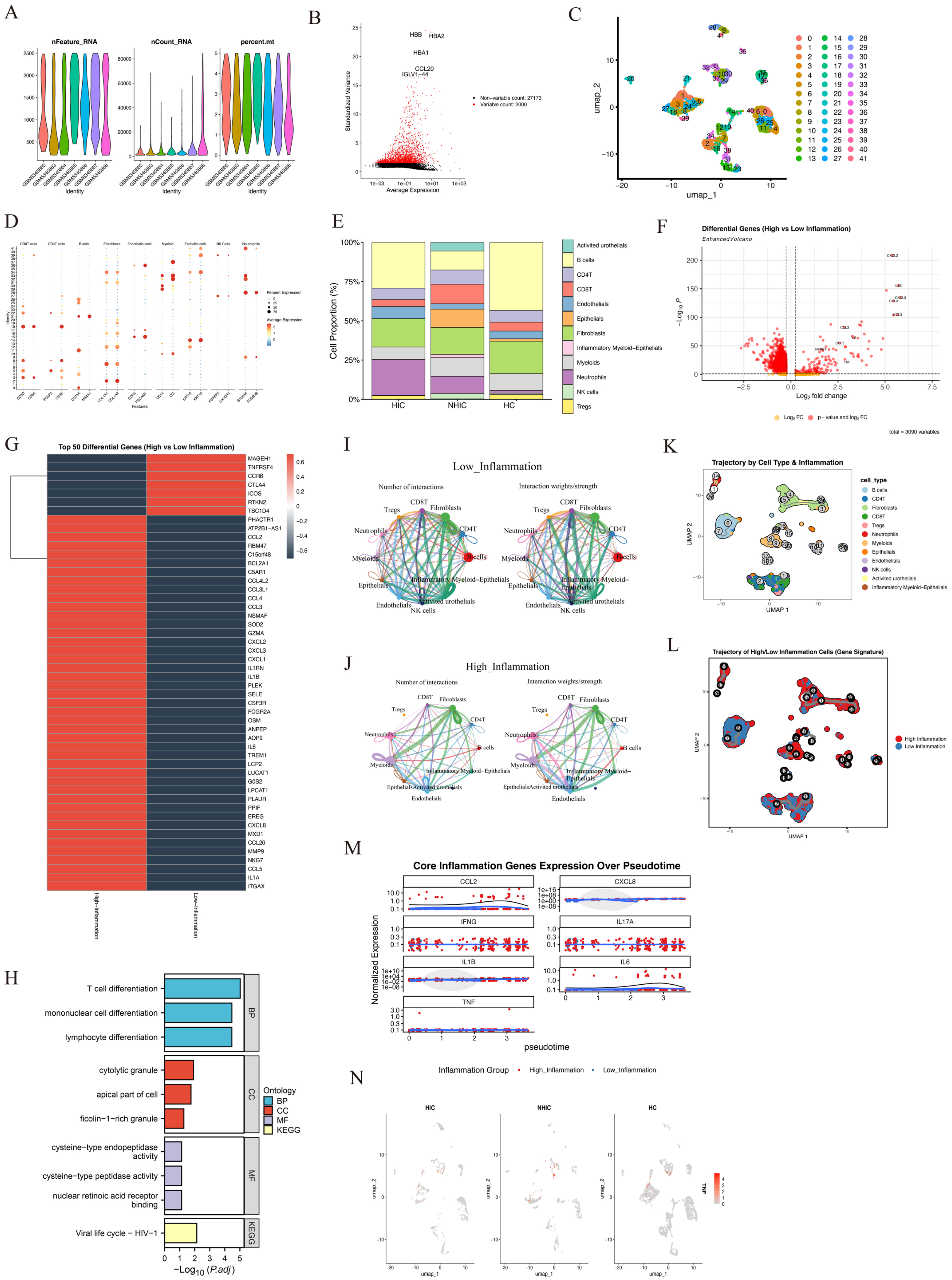

Supplement: Supplementary file 2 [file FSB2-40-e72173-s005.pdf]
