# Supplementary material for: Diagnostic Model Development for IC/BPS and Its Subtypes Using Clinical Indicators, Urinary Biomarkers, and Single‐Cell Transcriptomic Analysis
Source: FASEB J. 2026 Aug 4;40(15):e72173. doi: 10.1096/fj.202602743R (PMC13435336; doi:10.1096/fj.202602743R)

Figure S4 Correlation between inflammation score and TNF expression across bladder cell types in IC/BPS. Scatter plots demonstrating the p ositive correlation between inflammation score and TNF expression in multiple bladder cell populations, including Epithelials, Myeloids, $CD4^{+}$ T cells, NK cells, $CD8^{+}$ T cells, Tregs, Fibroblasts, Inflammatory Myeloid-Epithelials, Activated urothelia, B cells, Endothelials, and Neutrophils. Linear regression lines and 95% confidence intervals (CIs) are shown, with correlation coefficients (r) and *P*-values indicating statistical significance.

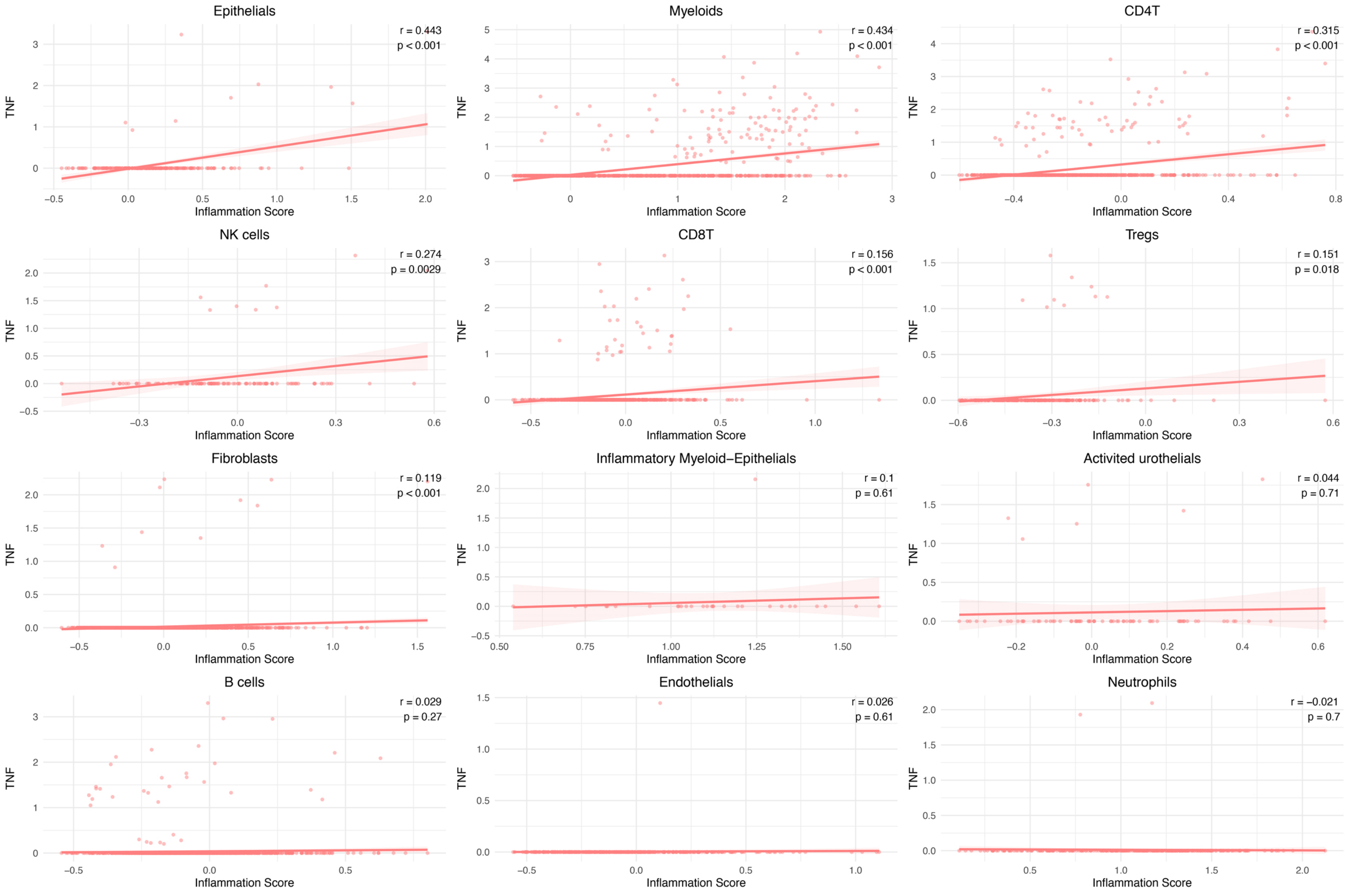

Supplement: Supplementary file 3 [file FSB2-40-e72173-s006.pdf]
