# Supplementary material for: Diagnostic Model Development for IC/BPS and Its Subtypes Using Clinical Indicators, Urinary Biomarkers, and Single‐Cell Transcriptomic Analysis
Source: FASEB J. 2026 Aug 4;40(15):e72173. doi: 10.1096/fj.202602743R (PMC13435336; doi:10.1096/fj.202602743R)

Figure S2 Transcriptomic analysis identifies distinct gene expression signatures in IC/BPS patients compared with HC. (A) Normalized signal intensities of differentially expressed probes in HC vs. IC/BPS and NHIC vs. HIC cohorts, demonstrating consistent upregulation. (B) PCA plots demonstrate clear separation between HC and IC/BPS patient groups, with PC1 and PC2 explaining the majority of the total variance. (C) Unsupervised hierarchical clustering of DEGs reveals distinct expression patterns in HC vs. IC/BPS and NHIC vs. HIC cohorts. Color scale indicates z-score-normalized expression levels: blue indicates downregulation and red indicates upregulation.

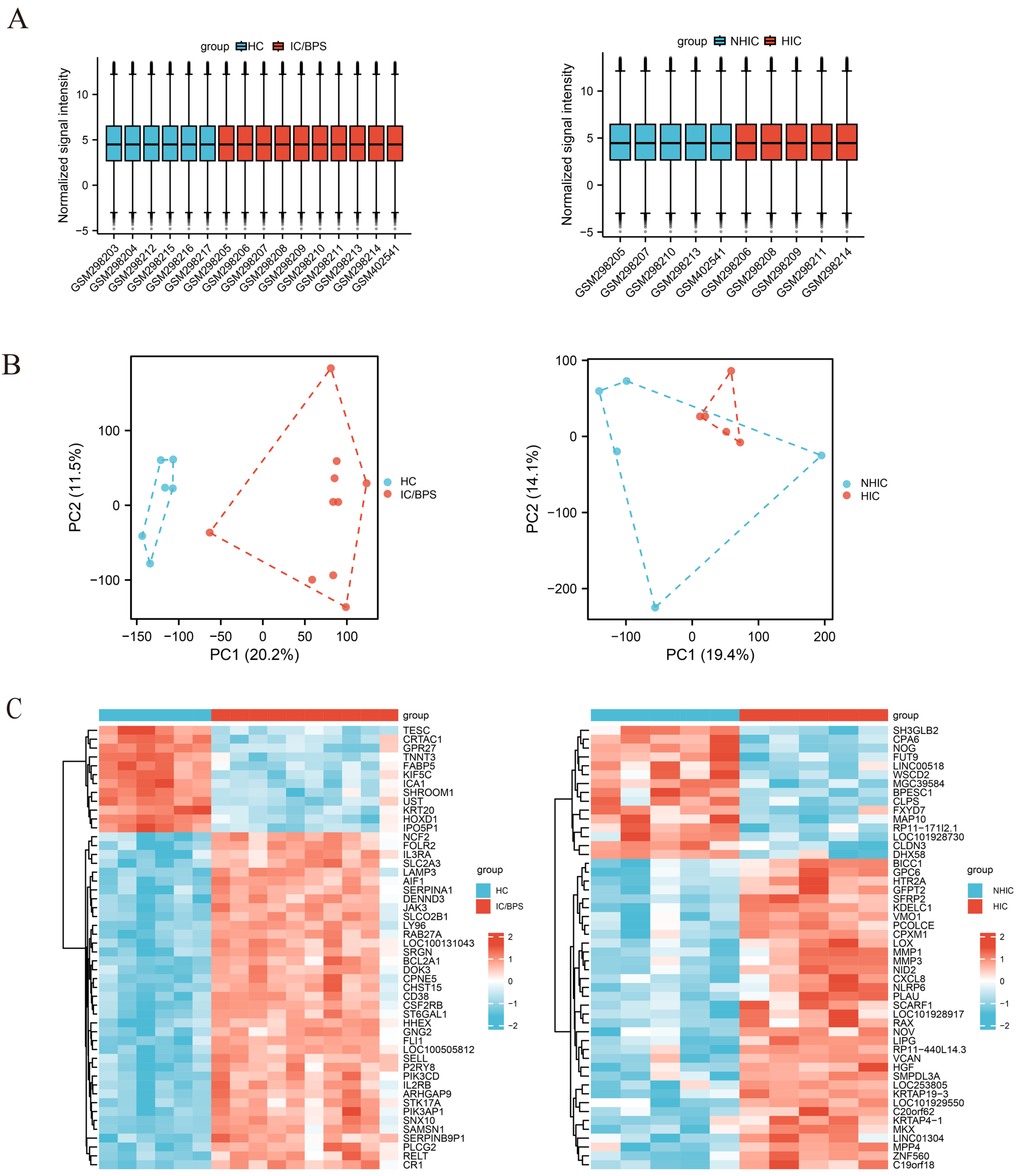

Supplement: Supplementary file 4 [file FSB2-40-e72173-s007.pdf]
