# Supplementary material for: Diagnostic Model Development for IC/BPS and Its Subtypes Using Clinical Indicators, Urinary Biomarkers, and Single‐Cell Transcriptomic Analysis
Source: FASEB J. 2026 Aug 4;40(15):e72173. doi: 10.1096/fj.202602743R (PMC13435336; doi:10.1096/fj.202602743R)
Supplement: Supplementary file 5 [file FSB2-40-e72173-s001.docx]

**Table S2** ROC analysis results of single indicators for distinguishing NHIC from HIC.

| **Indicators** | **AUC (95% CI)** | **Sensitivity (%)** | **Specificity (%)** |
| --- | --- | --- | --- |
| Clinical Characteristics |  |  |  |
| NUF | 0.986 (0.974–0.998) | 96.4 | 92.9 |
| Pelvic Pain VAS Score | 0.839 (0.784–0.894) | 73.2 | 81.6 |
| Urinary Ultrasound Parameters |  |  |  |
| FBC | 0.768 (0.696–0.839) | 76.8 | 76 |
| Functional BWT | 0.667 (0.570–0.764) | 55.4 | 88.3 |
| Urinary Biomarkers |  |  |  |
| IL-6 | 0.581 (0.502–0.659) | 75 | 48.5 |
| IL-8 | 0.537 (0.442–0.632) | 26.8 | 100 |
| TNF-α | 0.648 (0.555–0.741) | 50 | 87.2 |
| MCP-1 | 0.523 (0.441–0.606) | 89.3 | 21.4 |
| IP-10 | 0.487 (0.401–0.574) | 51.8 | 52 |
| MIP-1β | 0.537 (0.446–0.628) | 48.2 | 63.8 |
| RANTES | 0.545 (0.460–0.629) | 50 | 60.2 |
| 8-OHdG | 0.601 (0.516–0.685) | 21.4 | 100 |
| 8-iso-PGF2α | 0.611 (0.529–0.692) | 100 | 23 |
| TAC | 0.547 (0.461–0.633) | 26.8 | 85.2 |
| BDNF | 0.529 (0.441–0.617) | 78.6 | 30.1 |
| TGF-β | 0.584 (0.490–0.678) | 53.6 | 75 |
| Eotoxin | 0.605 (0.504–0.705) | 51.8 | 85.8 |
| NGF | 0.591 (0.507–0.675) | 85.7 | 34.2 |

**Abbreviations:** AUC, area under the curve; CI, confidence interval; NUF, nocturia frequency; VAS, visual analogue scale; FBC, functional bladder capacity; BWT, bladder wall thickness; IL‑6, interleukin‑6; IL‑8, interleukin‑8; TNF‑α, tumor necrosis factor‑α; MCP‑1, monocyte chemoattractant protein‑1; IP‑10, interferon‑γ‑inducible protein 10; MIP‑1β, macrophage inflammatory protein‑1β; RANTES, regulated on activation, normal T cell expressed and secreted; 8‑OHdG, 8‑hydroxy‑2’‑deoxyguanosine; 8-iso-PGF2α, 8-iso-Prostaglandin F2α; TAC, total antioxidant capacity; BDNF, brain‑derived neurotrophic factor; TGF‑β, transforming growth factor‑β; NGF, nerve growth factor.
