# Supplementary material for: Diagnostic Model Development for IC/BPS and Its Subtypes Using Clinical Indicators, Urinary Biomarkers, and Single‐Cell Transcriptomic Analysis
Source: FASEB J. 2026 Aug 4;40(15):e72173. doi: 10.1096/fj.202602743R (PMC13435336; doi:10.1096/fj.202602743R)
Supplement: Supplementary file 6 [file FSB2-40-e72173-s004.docx]

**Table S1** ROC analysis results of single indicators for distinguishing IC/BPS from HC.

| **Indicators** | **AUC (95% CI)** | **Sensitivity (%)** | **Specificity (%)** |
| --- | --- | --- | --- |
| Clinical Characteristics |  |  |  |
| NUF | 0.995 (0.991–0.998) | 93.3 | 98.3 |
| Pelvic Pain VAS Score | 0.992 (0.984–1.000) | 98.4 | 100 |
| Urinary Ultrasound Parameters |  |  |  |
| FBC | 0.933 (0.911–0.956) | 80.6 | 98.3 |
| Functional BWT | 0.975 (0.963–0.987) | 91.3 | 100 |
| Urinary Biomarkers |  |  |  |
| IL-6 | 0.632 (0.580–0.684) | 49.6 | 86.5 |
| IL-8 | 0.583 (0.532–0.634) | 24.6 | 96.1 |
| TNF-α | 0.858 (0.821–0.895) | 85.7 | 83.9 |
| MCP-1 | 0.527 (0.475–0.579) | 98 | 16.5 |
| IP-10 | 0.799 (0.758–0.841) | 57.5 | 73.5 |
| MIP-1β | 0.753 (0.711–0.796) | 68.7 | 97 |
| RANTES | 0.635 (0.586–0.684) | 60.3 | 78.7 |
| 8-OHdG | 0.739 (0.695–0.782) | 24.6 | 100 |
| 8-iso-PGF2α | 0.589 (0.538–0.641) | 68.3 | 77 |
| TAC | 0.799 (0.758–0.841) | 100 | 22.2 |
| BDNF | 0.520 (0.468–0.572) | 97.7 | 12.6 |
| TGF-β | 0.742 (0.695–0.789) | 62.3 | 100 |
| Eotoxin | 0.597 (0.546–0.648) | 97.7 | 23 |
| NGF | 0.793 (0.753–0.834) | 64.7 | 83.9 |

**Abbreviations:** AUC, area under the curve; CI, confidence interval; NUF, nocturia frequency; VAS, visual analogue scale; FBC, functional bladder capacity; BWT, bladder wall thickness; IL‑6, interleukin‑6; IL‑8, interleukin‑8; TNF‑α, tumor necrosis factor‑α; MCP‑1, monocyte chemoattractant protein‑1; IP‑10, interferon‑γ‑inducible protein 10; MIP‑1β, macrophage inflammatory protein‑1β; RANTES, regulated on activation, normal T cell expressed and secreted; 8‑OHdG, 8‑hydroxy‑2'‑deoxyguanosine; 8-iso-PGF2α, 8-iso-PGF2α, 8-iso-Prostaglandin F2α; TAC, total antioxidant capacity; BDNF, brain‑derived neurotrophic factor; TGF‑β, transforming growth factor‑β; NGF, nerve growth factor.
